# A review of Patient Reported Outcome Measures (PROMs) for characterizing Long COVID (LC)—merits, gaps, and recommendations

**DOI:** 10.1186/s41687-024-00773-1

**Published:** 2024-08-26

**Authors:** Hammed Ejalonibu, Adelaide Amah, Alaa Aburub, Pawan Kumar, D. E. Frederick, Gary Groot

**Affiliations:** 1 Saskatchewan Health Quality Council (HQC), Saskatoon, SK Canada; 2 Saskatchewan Center for Patient Oriented Research (SCPOR), University of Saskatchewan, Saskatoon, SK Canada; 3 Research Department, Saskatchewan Health Authority (SHA), Saskatoon, SK Canada; 4 Department of Community Health and Epidemiology, University of Saskatchewan, Saskatoon, SK Canada

**Keywords:** Long COVID (LC), Patient Reported Outcome Measures (PROMs), Merit matrix, Psychometric evidence, Health care utilization

## Abstract

**Background:**

Individuals may experience a range of symptoms after the clearance of the severe acute respiratory syndrome coronavirus 2 (SARS-CoV-2) infection. This condition is termed long COVID (LC) or Post-COVID-19 condition (PCC). Despite the appreciable number of symptoms documented to date, one key challenge remains in the robust characterization of LC outcomes. This review aimed to assess the properties, identify gaps, and provide recommendations for relevant descriptive and evaluative Patient-Reported Outcome Measurement (PROM) instruments that can be used to comprehensively characterize LC.

**Methods:**

To achieve this objective, we identified and reviewed descriptive and evaluative PROM instruments that have been developed and validated to date with people living with LC. Our review assessed their properties, identified gaps, and recommended PROMs suitable for characterizing LC. To ensure a comprehensive and robust characterization of LC, we next identified, reviewed, and selected (with the input of patient partners) PROMs associated with the most frequently reported LC symptoms. The evaluation criteria included psychometric evidence, mode of delivery, cost, and administration time.

**Results:**

Traditional matrix mapping revealed Post-COVID Functional Status Scale (PCFS) as a choice instrument for capturing LC outcomes largely because of the comprehensive domains it covered, and the number of psychometric evidence reported in literatures. This instrument can be effectively paired with the Fatigue Severity Scale (FSS), Montreal Cognitive Assessment (MoCA), Patient Health Questionnaire (PHQ-9), Headache Impact Test (HIT), Pittsburgh Sleep Quality Index (PSQI), and DePaul Symptom Questionnaire (DSQ-PEM) to characterize fatigue, cognitive impairment, depression/anxiety, headache, sleeplessness, and post-exertional malaise respectively.

**Conclusion:**

Our paper identified appropriate PROM instruments that can effectively capture the diverse impacts of LC. By utilizing these validated instruments, we can better understand and manage LC.

**Supplementary Information:**

The online version contains supplementary material available at 10.1186/s41687-024-00773-1.

## Plain language summary

Some individuals who once contracted the virus responsible for COVID-19 may continue to experience a range of symptoms persisting for more than 3 months. These symptoms include fatigue, difficulty sleeping, anxiety and difficulty breathing amongst others. This condition is known by many as Long COVID (LC). To understand the health outcomes of patients faced with this condition, standardized Patient Reported Outcome Measurement instruments (PROMs) are pivotal. PROMs are standardized questionnaires completed by patients to measure their symptoms, perceptions of health status, and/or functional well-being. In this paper, we have examined standardized instruments suitable for measuring LC outcomes. We conducted a comprehensive strength and weakness analysis of each instrument reviewed, guided by specific criteria. Based on our strength and weakness analyses, we identified several potential instruments that can be used to reports patients’ outcomes concerning LC. The data collected from PROMs provides valuable insights for healthcare practitioners and policy makers, enabling the enhancement of patient-centric care and serving as an instrument for systemic transformation.

**Supplementary Information:**

The online version contains supplementary material available at 10.1186/s41687-024-00773-1.

## Introduction

Evidence has shown that a range of symptoms can persist after the clearance of the acute SARS-CoV-2 infection phase in many individuals. This condition, termed long COVID (LC) or Post COVID Condition (PCC) and sometimes referred to as post-acute sequelae of SARS-CoV-2 infection (PASC) [1, 2], can last for weeks or months, resulting in further devastating effects on affected individuals.

The definition of LC varies slightly among different healthcare agencies. For example, the World Health Organization (WHO) defined LC as the “continuation or development of new symptoms three months after the initial SARS-CoV-2 infection, with these symptoms lasting for at least two months with no other explanation” [3]. On the contrary, the National Institute for Health and Care Excellence (NICE) defined LC “as the presence of signs and symptoms that develops during or after a COVID-19 episode, persisting for more than 12 weeks post-infection and cannot be explained by an alternative diagnosis” [4]. While there are slight variations in the definition of LC among recognized healthcare agencies, it is globally accepted that LC is a complex health condition that can affect individuals for an extended period following a recovery from the acute phase of COVID-19.

Studies have indicated that LC can impact multiple systems in the body [5, 6]. These studies describe the most affected systems as neurological, respiratory, cardiovascular, gastrointestinal, and musculoskeletal systems. Furthermore, the range of symptoms experienced by individuals with LC is extensive, including cognitive dysfunction, shortness of breath, cough, chest pain, and generalized muscle weakness to mention a few. These symptoms can significantly impact the physical functioning and quality of life (QOL) of affected individuals, leading to increased psychological distress. Despite the appreciable number of symptoms documented to date, newer symptoms are continuously being identified and added to the list as evidence emerges [7].

Patient-reported outcome measures (PROMs) are standardized questionnaires completed by patients to measure their symptoms, perceptions of health status, and/or functional well-being [8, 9]. If introduced as a system of measurement, PROMs can capture important information that can help clinicians and researchers to understand the impact of LC from the patients’ perspective. The data collected from PROMs provides vital insights for healthcare practitioners and policymakers, enabling the enhancement of patient-centric care and serving as an instrument for systemic transformation [10]. To elucidate, PROMs can enhance communication between patients and clinicians, leading to personalized care plans tailored to individual needs [11]. Furthermore, PROM data collected from a specific population and aggregated can help identify patterns and disparities in health outcomes, improving quality of care and policies within the healthcare system [12].

As new LC symptoms are reported, it is expected that standardized PROM instruments used to clinically assess patients will capture the most prevailing symptoms and treatment effects experienced by people with LC. A preliminary literature review identified four condition-specific PROM instruments [13, 14] for characterizing symptoms and assessing their impact on people with LC. However, none of these PROM instruments can be used as standalone measures to comprehensively capture the prevalent physical and psychological symptoms and assess the impact of these symptoms on daily life and functioning. This review aims to assess the different properties of some descriptive and evaluative patient reported outcome measurement instruments, identify gaps, and make recommendations on relevant PROM instruments that can be used to comprehensively characterize LC. Recognizing that no individual PROM tool can capture the vast array of individual symptoms reported on LC, it is most pragmatic for the LC research community to come to a consensus on which PROMs are best suited to characterize and assess the impacts of LC. This manuscript aims to critically evaluate the available condition-specific and symptoms specific PROM tools identified and contribute to this crucial decision-making process.

## Methodology

A literature review was conducted to identify PROM instruments developed and validated to date within the LC community. We reviewed and evaluated these instruments in two phases. First, we reviewed the different characteristics and dimensions of each instrument and documented any variations in the number of clinical domains covered. Next, we evaluated each instrument based on the following criteria: mode of administration, cost, average administration time, and validation of the tools. We utilized psychometric evidence (such as validity, reliability, and responsiveness) to assess the merits and gaps of these instruments. Based on the identified gaps, a second review was conducted to identify symptom-specific instruments that could be applied to measure symptoms of LC. The selection of additional symptom-specific instruments was informed by recommendations from people with lived experience using the previous criteria. Overall, our review used a systematic approach to compare the strengths and limitations of appropriate PROM instruments that can adequately capture the varied effect of LC.

### Information sources and extraction criteria

The information sources utilized for this review included PubMed/MEDLINE, Web of Science, Scopus, APA PsycINFO, Google Scholar, and the Australian Commission on Safety and Quality in Healthcare databases. The literature searches in these databases were conducted between January 31, 2023, and January 30, 2024. We included all English language articles, case series, and review articles on PROMs for LC. Additionally, we included articles with neurological, respiratory, cardiovascular, gastrointestinal, and musculoskeletal PROMs. In this review, we have excluded articles that were not written in English language, and articles with PROMs reported by proxy. The first author (H.E.) searched for the available PROM instruments. Following the initial search, H.E. reviewed and evaluated the PROM instruments for their psychometric properties. All other authors were asked to review this work to ensure reliability of the process.

### Data extraction and quality assessment

The data extraction process was carried out by the first author (H.E.) and again reviewed by all authors. Data extraction was performed followed a traditional merit matrix developed for this review, which was approved by all authors. The merit matrix included criteria such as psychometric evidence, mode of delivery, average administration time and resources required to administer the tool, and the language in which the tool was validated amongst others.

### Patient partner engagement

In this study, we consulted patient partners with lived experiences of LC. These individuals actively participated in a collaborative process to select symptom-specific PROM instruments based on the mode of administration, cost, average administration time, and validation of the tools. These criteria were agreed upon by the authors and patient partners based on combined expertise, and a review of parameters commonly used in literatures. Patient partners also identified potential barriers for patients using these tools (considering factors such as language, readability, ease of use and/or feasibility for completion.), drawing on their own experience of the condition. To select complementary tools for monitoring specific symptoms in LC patients, we compared over 27 PROM instruments measuring fatigue, sleep disturbances, post brain fog, headache, difficulty breathing, cough, dizziness, post-exertional malaise (PEM), and widespread musculoskeletal pain with the assistance of patient partners. Their insights were instrumental in identifying tools that encompass outcomes that matter the most to patients.

## Results

Our search identified four condition-specific PROM instruments available for assessing outcomes related to LC: the Post-COVID Functional Status Scale (PCFS), the Symptom Tool and Impact Tool (ST & IT), the Symptom Burden Questionnaire (SBQ™– LC), and the COVID-19 Yorkshire Rehabilitation Scale (C19 -YRS). These instruments cover a wide range of domains relevant to LC, including physical functioning, emotional well-being, functional limitations, social interactions, and some other disease-specific aspects.

### Description of instruments

#### Post-COVID Functional Status Scale (PCFS)

The Post-COVID Functional Status Scale (PCFS) was designed to assess the functional status and limitations of individuals experiencing LC [13]. This PROM instrument captures the impact of LC on various aspects of daily functioning and overall well-being. The PCFS consists of questions that cover different domains, such as physical abilities, cognitive functioning, emotional well-being, and social interactions [15]. The PCFS a descriptive tool is ordinal, featuring five numerical steps ranging from grade 0 (no symptoms) to 4 (severe functional limitations), covering a diverse number of functional outcomes and challenges faced by individuals suffering from LC. This scale is simple yet powerful, as it helps in gathering valuable information about the functional outcomes of LC.

#### Symptom Burden Questionnaire (SBQ™– LC)

The Symptom Burden Questionnaire (SBQ™– LC) is used to assess the severity and impact of symptoms experienced by individuals with LC [16]. The impact aspect of the SBQ™– LC measures how severely and significantly the symptoms associated with LC affect various aspect of an individual’s life. The burden aspect refers to the subjective experience the symptoms impose on individuals living with LC; in essence it measures the difficulty and limitations causes because by these symptoms. The construct of measure here is focused on understanding the perceived severity, impact, and burden of the symptoms experienced by individuals living with LC. This comprehensive questionnaire is comprised of 123 subjective questions. The instrument includes a range of questions that cover various symptoms of LC, such as pain, breathlessness, vascular circulation, and general well-being, among others. In addition, the tool measures the overall symptom burden. This reported outcome instrument allows patients to identify the severity (none, grade 0 to severe, grade 4) and frequency of symptoms (never, grade 0 to always, grade 4) experienced on a rating and dichotomous scale. The SBQ™– LC is a robust descriptive instrument with 17 different domains and 123 questions, and data for validation was obtained remotely on social media platforms in the United Kingdom.

#### Symptom Tool and Impact Tool (ST & IT)

The Symptom Tool and Impact Tool (ST & IT) are validated, and reliable patient-reported instruments developed for monitoring the symptoms and impact of LC disease on patients [17]. Like other tools earlier discussed, the ST & IT tool aims to provide a comprehensive understanding of the symptoms experienced by individuals with LC and assess how these symptoms affect their lives. The symptom and impact tools, although they are often used together in the research context, they are used to report different measures. For example, the symptom tool captures and quantifies changes in specific symptoms associated with LC over a period of 30 days. On the other hand, the impact tool aims to evaluate the broader impact of LC on patients’ lives by assessing questions related to physical functioning, emotional well-being, and overall social interactions. There are several constructs this instrument measures which are symptoms, impact on daily life, quality of life and functional impairment. This ST & IT is a descriptive PROM tool constructed from answers to surveys with open-ended questions delivered to 492 patients.

#### COVID-19 Yorkshire Rehabilitation Scale (C19-YRS)

The COVID-19 Yorkshire Rehabilitation Scale (C19-YRS) measures the overall impact of LC on health status [14]. It is a global health questionnaire used to record patient symptoms, functioning, and disability. There are several constructs this instrument measures which are physical functioning, respiratory symptoms, psychological impact, cognitive functioning, impact on daily life, quality of life and functional impairment. This tool is a 22-item PROM with symptom severity (0-100), functional disability (0–50), additional symptoms (0–60), and overall health (0–10) scales.

Table 1 displays the primary characteristics of the selected condition-specific PROM instruments, including the mode of administration, resources needed to administer the tool, response options, range of scores, average administration time, and original language of validation.

In Table 2, we have examined the breadth of domains captured by the selected condition-specific PROM instruments. Our choice of domains was informed by the World Health Organization’s International Classification. These were categorized as: physical functioning, emotional well-being, social functioning, cognitive functioning, other symptoms, health-related quality of life (HRQOL), and disease-specific domains.

### Quality evaluation

In Table 3, the quality of the condition-specific PROM instrument was evaluated using three critical criteria: reliability, validity, and responsiveness. These criteria serve as essential benchmarks for assessing the robustness and suitability of PROMs in capturing patient experiences and outcomes. Given these quality criteria, in Table 3, we ranked all instruments based on the number of psychometric properties that was found to have been reported in literatures on a scale from 1 to 7.

**Table 1:** Characteristics of selected long COVID (LC) instruments: the mode of administration, number of participants used for initial studies, response options, range of scores, average administration time and the original language of validation

Instrument	Summary of tool	Mode of administration	Purpose	Participants	Response options	Range of scores	Average administration time	Original language
Post-COVID Functional Status Scale (PCFS) [13]	Designed to identify rehabilitation needs of patients with LC. Questions aim to identify how much the patient is currently affected in their daily life by COVID-19. It has 4 items.	Paper and can be delivery electronically	Descriptive	1939	Yes/No	0–4	5–10 min	English
Symptom and Impact Tool (ST & IT) [17]	Developed to monitor symptoms and impact of LC on patients’ lives. It has 53 items in total.	Paper	Descriptive	492	–	–	5–15 min	French
Symptom Burden Questionnaire (SBQ™– LC) [16]	Measures symptom impact on daily life. Comprised of 17 independent scales covering 16 symptom domains and 1 scale measuring symptom impact on daily life. It has 123 items in total.	Paper delivery but can be administered electronically	Descriptive	274	None/Mild/Moderate/Severe	1–4	20–30 min	English
					Never/Rarely/Sometimes/Always			
					Yes/No			
COVID-19 Yorkshire Rehabilitation Scale (C19-YRS) [14]	Records patients’ symptoms, functioning, and disability. It consists of 22 items.	Paper delivery but can be administered electronically	Descriptive	187	None/Mild/Moderate/Severe	0–3	–	English

**Table 2:** Domains reported in the selected instruments

Instrument	Domains						
	Physical	Emotional	Social	Cognitive	Symptoms	Health-related Quality of Life (HRQOL)	Disease-specific domains
PCFS	+	+	+	+	--	+	--
ST & IT	--	+	+	+	+	+	--
SBQ™– LC	+	+	+	+	+	+	--
C19-YRS	+	+	+	+	+	+	--

‘+’ indicates domains that are present in the PROM instrument, while ‘--’ indicates domains that are not present in the PROM instrument reviewed

**Table 3:** Quality assessment of all identified PROM instrument

Instrument	Reliability		Validity				Responsiveness	Score
	Internal consistency	Test-retest	Face validity	Content validity	Criterion validity	Construct validity		
PCFS [18]	$\:\alpha\:\:=\:0.821$	ICC = 0.821	+	+	+	+	+	7/7
ST & IT [17]	$\:\alpha\:\:=\:0.75\:-\:0.95$	$\:r\:=0.35-\:0.50$	+	?	?	+	?	4/7
SBQ™– LC [16]	$\:\alpha\:\:=\:0.56\:-\:0.91$	?	+	+	+	+	?	5/7
C19-YRS [14]	$\:\alpha\:\:=\:0.891$	$\:r\:=-0.218-\:0.772$	+	?	?	+	+	5/7

$\:\alpha\:$ is the Cronbach’s Alpha co-efficient used to measure internal consistency. ICC (intra-class correlation co-efficient) and $\:r\:$(Spearman rank correlation coefficient) measures test-retest reliability. ‘+’ indicates measurement property that was reported, while ‘?’ indicates measurement properties that was not reported as of the date this paper was published

## Discussion

In this review, we identified and compared condition-specific PROM instruments developed to characterize LC. The direct comparison of these instruments took into consideration parameters such as the characteristics of the tool, its method of administration (including resources needed), average administration time, number of domains included, how the tool was validated in the population (including the language of validation), as well as psychometric properties related to its reliability, validity and responsiveness.

### Method of administration and domains

Considering that the method of delivery when administering PROMs is important as this can considerably impact accessibility and patient engagement. We extracted this information for our analysis of the tools because we anticipated that the PROM tools used to characterise LC would predominantly be administered electronically for ease of use, and to facilitate rapid knowledge sharing between patients and healthcare providers. This option is also convenient for patients and caregivers, leading to better participation. All instruments are available for both electronic and paper delivery, except for the ST & IT tool which has only been administered on paper. On the domain aspect, all four PROM tools compared reported physical, emotional, social, cognitive, symptoms and health-related quality of life domains except for PCFS where the symptoms domain is not present, and ST & IT which does not include the physical domain. None of these instruments has a disease-specific domain, which is usually tailored to the needs and experiences of patients. Therefore, from our analysis, it appears that all the instruments reviewed lacked one or more domains and cannot be used as standalone measures to comprehensively capture all the prevalent physical and psychological symptoms within this population. Interestingly, for the ST & IT instrument, the symptoms domain constitutes more than 70% of the entire instrument, with a significantly small percentage of impact-related questions. This instrument is thus over-weighted towards the symptom’s domain.

### Validation within the population

Of the four instruments, the PCFS utilized the highest number of participants during its initial validation phase (*n* = 1939), compared to the ST & IT instrument with *n* = 1022, and the C19-YRS with *n* = 187 participants respectively. Using a large population for validation indicates rigorous sample testing. For the SBQ™, data for validation was obtained remotely on social media platforms in the United Kingdom. This means that there is no guarantee for the generalizability of this tool as it might not be effective and applicable to a different population or in different cultural settings. Considering the ST & IT instrument, validation occurred with a diverse audience of 1022 participants, regardless of the number of participants that took part in the validation of these instrument, concerns about whether the ST & IT tool covers a full spectrum of symptoms and questions about the obstacles and constraints experienced by people living with LC remains.

O’Connor et al. [14] conducted a study to evaluate the clinical usefulness and psychometric properties of the C19-YRS using 187 patients attending a COVID-19 rehabilitation clinic. The results indicate that the C19-YRS is clinically useful and satisfies standard psychometric criteria, thus providing initial evidence of its suitability to characterize LC.

Building on this work, a similar study by Sivan et al. [19] tested the C19-YRS scale’s psychometric properties using Rasch analysis and modified the scale based on feedback from a working group of about 370 patients and professionals. Post hoc rescoring suggested that a 4-point response category structure would be more appropriate than an 11-point response subscale in the previous C19-YRSm. Based on these findings, a 17-item modified COVID-19 Yorkshire Rehabilitation Scale (C19-YRSm) was developed with subscales (scores); symptoms severity (0–30), functional disability (0–15), other symptoms (0–25), and overall health (0–10). The C19-YRS validation is ongoing, thus limiting its generalizability.

### Language

Regarding the original languages of development, all the instruments were developed in English, except for the ST & IT tool which was originally developed in French. Further comparison of PROM instruments in Table S1 shows that the PCFS and the C19-YRS have been validated in multiple languages, in comparison to their ST&IT and SBQ™– LC counterparts, which have only been validated in one language [20]. For instance, the PCFS and C19-YRS offer questions and statements translated and validated in various languages such as English, Spanish, and Mandarin, whereas the ST&IT and SBQ™– LC are only available in English. This is important to highlight because outcome measures validated in a greater number of languages have more relevance, applicability, cultural sensitivity, and accessibility. Acquadro et al. [21] demonstrated that PROMs validated in multiple languages offer broader cultural representation and capture diverse perspectives on health-related quality of life compared to those validated in only one language. Similarly, research by Wild et al. [20] highlighted how linguistic and cultural adaptation of PROMs ensures the conceptual equivalence of questions across languages, thus facilitating accurate measurement and interpretation of outcomes in various cultural settings. For example, in this context, different cultures and language backgrounds can have unique perspectives on LC and its corresponding quality of life. Validating PROM tools in multiple languages ensures that the intended meaning and relevance of the questions are captured in diverse cultural settings. Importantly, from a patient-centered care perspective, translating and validating a PROM tool in multiple languages can help patients fully understand and provide accurate and meaningful responses. As a rule of thumb, outcome measures validated in a greater number of languages have more relevance, applicability, cultural sensitivity, and accessibility. Furthermore, validating PROMs in multiple languages allows for the comparison of results internationally, hence increasing the generalizability and robustness of outcome results [22].

### Average administration time

In terms of administration time and the resources required to administer each tool, the literatures examined affirm that all instruments reviewed have an average administration time ranging between 5 and 30 min. Using a simple and short but robust PROM tool are essential to capture relevant comprehensive information while increasing the response rate and reducing the burden on patients. This allows for a meaningful and efficient assessment of the patient experience. Furthermore, shorter PROM questionnaires require fewer resources for administration, making them cost-effective for researchers and healthcare providers. In clinical LC settings, the use of short and simple PROMs will benefit LC patients, especially those experiencing brain fog or other challenging cognitive deficits who may struggle to complete longer PROM tools. Given this, we deduced that the PCFS and the ST & IT instruments can be administered within a shorter period than the C19-YRSm and SBQ™– LC; this makes them a choice tool above others.

### Psychometric evidence

The most frequently reported properties are reliability (internal consistency, test-retest reliability), validity (construct validity and face validity) and responsiveness. The psychometric properties are quality assurance measures put in place to ensure confidence in the data obtained from patients. Internal consistency is usually presented as Cronbach’s $\:\alpha\:$ with values of over 0.70, and this property gauges how well the item within an instrument collates with each other. Responsiveness on the other hand reflects how a PROM instrument detects meaningful changes in a patient’s health status. In comparing all four tools we found that responsiveness was only reported for the PCFS and the C19-YRS tools. Next, considering the validity of all the instruments examined, only the PCFS and the SBQ™– LC were reported in the literature to have been content and criterion-validated. This validation underscores the PCFS and the SBQ™– LC instrument’s suitability for assessing specific life changes. When ranked, the PCFS has a perfect quality score (7/7) across the three measurement properties (validity, reliability, and responsiveness), while the ST & IT instrument has the lowest score (4/7) with some measures of validity (content validity, criterion validity) and responsiveness not reported. Understanding the psychometric properties of a PROM tool ensures that the tool is valid, reliable, interpretable, and suitable for use in a clinical or research setting [23].

## Our recommendation

Based on these analyses, we selected the PCFS as a tool that can be used to adequately capture outcomes in LC patient populations. From our analysis, the PCFS stands out as a tool that satisfies all the properties of interest, however, one drawback is that this instrument provides a broad classification of functional status levels and would not be able to capture nuanced changes or specific aspects of functional limitations that individuals may experience. This instrument also does not cover the full range of symptoms or specific impairments experienced by individuals living with LC. Since the symptoms experienced by LC patients vary, we believe it is best to adopt one instrument that examines general and functional limitations while accommodating additional symptom-specific instruments to capture other aspects of the LC patient experience.

**Fig. 1:**
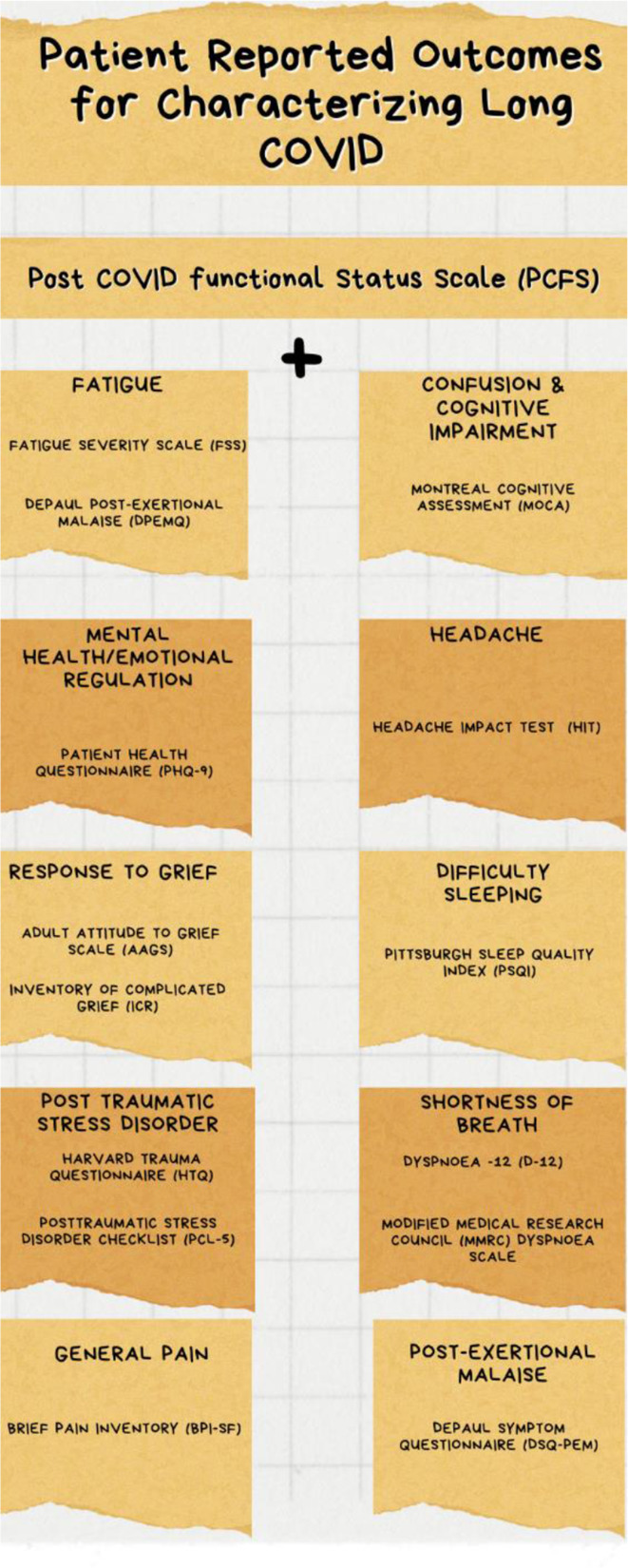
Lists of selected symptom specific tools for characterizing LC

Based on this comparison, we were able to identify fourteen additional PROM instruments that can be used to characterize LC symptoms more comprehensively, as shown in Fig. 1 (and in Table S2). Our findings do not suggest that the other PROMs outlined are unacceptable instruments for capturing LC symptoms; this selection was purely based on the relevance, acceptability, simplicity, and convenience of administration of the selected PROM instruments by patient partners. We also acknowledge that a combination of different PROM tools may be needed to ensure that the relevant domains are captured based on new findings in the literature.

There are limitations to this review. First, in characterizing symptoms associated with LC, we have only included the top symptoms reported by Statistics Canada. This list of symptoms represents a fraction of what has been reported, as there are an appreciable number of symptoms that have been documented to date. For a robust characterization, the identification of PROMs for other LC symptoms not accounted for in this review is needed. Secondly, we have identified and selected LC condition-specific instruments based on specific parameters. These parameters were subjectively determined by the authors and a few patient partners. Authors and patient partners agreed to select tools based on a few criteria. Tool selection and identification could benefit from other methods of gaining consensus, such as utilizing the Delphi approach with a group of clinicians, policymakers, clinicians, and patients with lived experience.

While the current set of PROMs included in this review provides a foundational framework for characterizing LC, it is crucial to recognize that the choice of the PROMs instrument will vary depending on the aim of the study. For instance, our review aims to identify tools to characterize LC and hence the reason why we have been confined to descriptive and some evaluative PROM instruments only.

## Conclusion

Our review identified descriptive Patient-Reported Outcome Measures (PROMs) to characterize symptoms and assess outcomes in people living with LC. Our review was conducted in two phases. First, PROM instruments developed and validated to date within the LC community were reviewed and evaluated. This evaluation considered psychometric evidence, mode of delivery, cost, and administration time to analyze the merits and gaps of the instruments. Based on the identified gaps, additional LC symptoms-specific tools were examined. The selection of additional symptom-specific tools was informed by recommendations from patient partners. The review used a systematic approach to find appropriate PROM tools that can adequately capture the varied effects of LC. Multi-comparison of LC-specific instruments identified the Post-COVID Functional Status Scale (PCFS) as a quality tool used to capture outcomes in the LC patient population. To further characterize LC symptoms, this instrument can be effectively paired with the Fatigue Severity Scale (FSS), Montreal Cognitive Assessment (MoCA), Patient Health Questionnaire (PHQ-9), Headache Impact Test (HIT), Pittsburgh Sleep Quality Index (PSQI), and DePaul Symptom Questionnaire (DSQ-PEM) to characterize fatigue, cognitive impairment, depression/anxiety, headache, sleeplessness, and post-exertional malaise respectively.

Using this approach, our review identifies appropriate descriptive PROM instruments that can effectively capture the diverse impacts of LC. The data collected from these identified PROM tools will provide vital insights for healthcare practitioners and policymakers, enabling the enhancement of patient-centric care and serving as an instrument for transformation in LC healthcare reporting. Additionally, this work can be used a framework to guide the selection of outcomes measures related to LC in clinical practice.

### Electronic supplementary material

Below is the link to the electronic supplementary material.

Supplementary Material 1

## Data Availability

All data and materials are available for circulation on the request from the corresponding author.

## References

[1] Callard F, Perego E (2021) How and why patients made Long Covid. Soc Sci Med 268:113426. 10.1016/j.socscimed.2020.113426 33199035 10.1016/j.socscimed.2020.113426 PMC7539940

[2] Baig AM (2021) Chronic COVID syndrome: need for an appropriate medical terminology for long-COVID and COVID long-haulers. J Med Virol 93(5):2555–2556. 10.1002/jmv.26624 33095459 10.1002/jmv.26624

[3] WHO Team. Global COVID-19 Clinical Platform Case Report Form (CRF) for Post COVID condition (Post COVID-19 CRF). https://www.who.int/publications/i/item/global-covid-19-clinical-platform-case-report-form-(crf)-for-post-covid-conditions-(post-covid-19-crf). Accessed 12 Jul 2024

[4] Shah W, Hillman T, Playford ED, Hishmeh L (2022) Managing the long term effects of covid-19: summary of NICE, SIGN, and RCGP rapid guideline. BMJ. Accessed 12 Jul 2024 https://www.bmj.com/content/376/bmj.o126 10.1136/bmj.n136 33483331

[5] Michelen M, Manoharan L, Elkheir N et al (2021) Characterising long COVID: a living systematic review. BMJ Glob Health. Accessed 12 Jul 2024 https://gh.bmj.com/content/6/9/e005427.abstract 10.1136/bmjgh-2021-005427 PMC8478580 34580069

[6] Quinn KL, Razak F, Cheung AM (2023) Diagnosing post-COVID-19 condition (long COVID) in adults. CMAJ. Accessed 12 Jul 2024 https://www.cmaj.ca/content/195/2/E78 10.1503/cmaj.220818 PMC9851644 36649955

[7] Raj SR, Arnold AC, Barboi A et al (2021) Long-COVID postural tachycardia syndrome: an American Autonomic Society statement. Clin Auton Res 31(3):365–368. 10.1007/s10286-021-00798-2 33740207 10.1007/s10286-021-00798-2 PMC7976723

[8] McKercher JP, Slade SC, Jazayeri JA et al (2022) Patient experiences of codesigned rehabilitation interventions in hospitals: a rapid review. BMJ Open. https://bmjopen.bmj.com/content/12/11/e068241.abstract. Accessed 12 Jul 2024 10.1136/bmjopen-2022-068241 PMC9639115 36332956

[9] Brusco NK, Atkinson V, Woods J et al (2022) Implementing PROMS for elective surgery patients: feasibility, response rate, degree of recovery and patient acceptability. J Patient-Rep Outcomes 6(1):73. 10.1186/s41687-022-00483-6 35798915 10.1186/s41687-022-00483-6 PMC9263014

[10] Kynoch K, Ameen M, Ramis MA, Khalil H (2022) Use of patient-reported data within the acute healthcare context: a scoping review. Int J Environ Res Public Health 19(18):11160. 10.3390/ijerph191811160 36141433 10.3390/ijerph191811160 PMC9517657

[11] Greenhalgh J, Gooding K, Gibbons E et al (2018) How do patient reported outcome measures (PROMs) support clinician-patient communication and patient care? A realist synthesis. J Patient-Rep Outcomes 2(1):42. 10.1186/s41687-018-0061-6 30294712 10.1186/s41687-018-0061-6 PMC6153194

[12] Greenhalgh J (2009) The applications of PROs in clinical practice: what are they, do they work, and why? Qual Life Res. https://link.springer.com/article/10.1007/s11136-008-9430-6. Accessed 12 Jul 2024 10.1007/s11136-008-9430-6 19105048

[13] Klok FA, Barco S, Endres M et al (2020) The Post-COVID-19 Functional Status scale: a tool to measure functional status over time after COVID-19. Eur Respir J. https://erj.ersjournals.com/content/56/1/2001494.short. Accessed 12 Jul 2024 10.1183/13993003.01494-2020 PMC7236834 32398306

[14] O’Connor RJ, Preston N, Parkin A et al (2022) The COVID-19 Yorkshire Rehabilitation Scale (C19-YRS): application and psychometric analysis in a post-COVID-19 syndrome cohort. J Med Virol 94(3):1027–1034. 10.1002/jmv.27415 34676578 10.1002/jmv.27415 PMC8662016

[15] Benkalfate N, Eschapasse E, Georges T et al (2022) Evaluation of the Post-COVID-19 functional status (PCFS) scale in a cohort of patients recovering from hypoxemic SARS-CoV-2 pneumonia. BMJ Open Respir Res 9(1):e001136. 10.1136/bmjresp-2021-001136 35264326 10.1136/bmjresp-2021-001136 PMC8915286

[16] Hughes SE, Haroon S, Subramanian A et al (2022) Development and validation of the symptom burden questionnaire for long covid (SBQ-LC): Rasch analysis. BMJ 377:e070230. 10.1136/bmj-2022-070230 35477524 10.1136/bmj-2022-070230 PMC9043395

[17] Tran VT, Riveros C, Clepier B et al (2022) Development and Validation of the Long Coronavirus Disease (COVID) symptom and impact tools: a set of patient-reported instruments constructed from patients’ lived experience. Clin Infect Dis. Oxford Academic. Accessed 12 Jul 2024 https://academic.oup.com/cid/article/74/2/278/6252414 10.1093/cid/ciab352 PMC8135558 33912905

[18] Kütükcü E, Çakmak A, Kinaci E et al (2021) Reliability and validity of the Turkish version of Post-COVID-19 functional status scale. Turk J Med Sci 51(5):2304–2310. 10.3906/sag-2105-125 34392673 10.3906/sag-2105-125 PMC8742502

[19] Sivan M, Preston N, Parkin A et al (2022) The modified COVID-19 Yorkshire Rehabilitation Scale (C19‐YRSm) patient‐reported outcome measure for Long Covid or Post‐COVID‐19 syndrome. J Med Virol. Wiley Online Library. https://onlinelibrary.wiley.com/doi/full/10.1002/jmv.27878. Accessed 12 Jul 2024 10.1002/jmv.27878 PMC9348420 35603810

[20] Wild D, Grove A, Martin M et al (2005) Principles of good practice for the translation and cultural adaptation process for patient-reported outcomes (PRO) measures: report of the ISPOR Task Force for Translation and Cultural Adaptation. Value Health 8(2):94–104. 10.1111/j.1524-4733.2005.04054.x 15804318 10.1111/j.1524-4733.2005.04054.x

[21] Acuqadro C, Conway K, Giroudet C, Mear I (2004) Linguistic validation Manual for patient-reported outcomes (PRO) Instruments. MAPI

[22] Lin MP, Kligler SK, Friedman BW et al (2023) Barriers and best practices for the use of patient-reported outcome measures in emergency medicine. Ann Emerg Med 82(1):11–21. 10.1016/j.annemergmed.2022.12.017 36682996 10.1016/j.annemergmed.2022.12.017 PMC10293024

[23] Frost MH, Reeve BB, Liepa AM, Stauffer JW, Hays RD, The Mayo/FDA Patient-Reported Outcomes Consensus Meeting Group (2007). What is sufficient evidence for the reliability and validity of patient-reported outcome measures? Value Health. Wiley Online Library. https://onlinelibrary.wiley.com/doi/full/10.1111/j.1524-4733.2007.00272.x. Accessed 12 Jul 2024 10.1111/j.1524-4733.2007.00272.x 17995479

