# Supplementary material for: A review of Patient Reported Outcome Measures (PROMs) for characterizing Long COVID (LC)—merits, gaps, and recommendations
Source: J Patient Rep Outcomes. 2024 Aug 26;8:101. doi: 10.1186/s41687-024-00773-1 (PMC11347522; doi:10.1186/s41687-024-00773-1)
Supplement: Supplementary file 1 — Supplementary Material 1 [file 41687_2024_773_MOESM1_ESM.docx]

| **Tool** | **Validation Language** | **Administration time & Resources** | **Psychometric Properties** | **Mode of delivery** |
| --- | --- | --- | --- | --- |
| PCFS | English, Spanish, Italian, French, and Dutch | Short and Simple | Reliability, validity, responsiveness, and MCID | Can be administered electronically |
| ST&IT | French | More time to administer than the PCFS and C19-YRS | Reliability, validity, and patient acceptable symptomatic state | Paper |
| SBQ^TM^ – LC | English | Relatively longer and takes more time to administer | Reliability and validity | Can be administered electronically |
| C19 -YRS | English, Thai, and Chinese | Short and Simple | Reliability, validity, and MCID | Can be administered electronically |

**Table S1:** Multi-comparison of LC condition specific PROM tools on a merit-matrix. This merit-matrix used colour coding to identify desired properties. For example, properties coloured “dark green” are “highly desired”, while those colored “red” are “not of interest” given the parameters listed above. The PCFS satisfies all properties sought after in this study.

**Table S2:** Lists of some condition-specific symptoms related to LC, identified collaboratively by authors and patient partners

| **Tool** | **Summary of Tool** | **No of Questions** | **Validity** | | **Purpose** | | | | | **Participants** | | | | | **Mode of Delivery** |
| --- | --- | --- | --- | --- | --- | --- | --- | --- | --- | --- | --- | --- | --- | --- | --- |
| **Fatigue** |  |  |  | |  | | | | |  | | | | |  |
| Fatigue Severity Scale (FSS) | A 9-item scale which measures the severity of fatigue and its effect on a person's activities and lifestyle in patients with a variety of disorders. Intended population are those with a variety of diagnoses including arthritis, stroke, and COVID-19 | 9 | Internal consistency, test-retest reliability, and validity in a range of clinical populations | | Descriptive | | | | | 74 | | | | | Can be administered electronically |
| DePaul Post-Exertional Malaise (DPEMQ) | The DePaul Post-Exertional Malaise Questionnaire or DPEMQ is a 53-item questionnaire designed to assess [post-exertional malaise](https://me-pedia.org/wiki/Post-exertional_malaise). Sections included in this questionnaire include demographics and illness information, onset and triggers, consequences and symptoms, duration, recovery, and pacing. | 53 | Construct validity, content validity, criterion validity, test-retest reliability, internal consistency, and factor analysis. | | | Descriptive | | | | 89 | | | | | Administered on paper and pencil – possibility to administer electronically with permission. |
| **Confusion and Cognitive Impairment** |  |  |  | | |  | | | |  | | | | |  |
| Montreal Cognitive Assessment (MoCA) | MoCA assesses cognitive impairment and has been validated in various patient populations. It consists of 30 items that evaluate various cognitive domains, such as attention, memory, language, and visuospatial abilities. This instrument is a sensitive measure of cognitive impairment. | 30 | Cross-sectional comparisons, longitudinal follow-up, correlation with neuroimaging measures, and comparison with other cognitive screening tools. | | | Evaluative | | | | 183 | | | | | Can be administered electronically |
| **Mental Health / Emotional Regulation** |  |  |  | | |  | | | |  | | | | |  |
| Patient Health Questionnaire (PHQ-9) | The PHQ-9 is a brief psychological screening instrument designed to measure symptoms of depression in primary care settings. | 9 | Construct validity, and criterion validity | | | Evaluative | | | | 6000 | | | | | Can be delivered electronically. It is available in a variety of formats, including online, paper, and telephone. |
| Patient Reported Outcomes Measurements Information Systems (PROMIS) | The PROMIS is one of the most widely used of the tools in the PROMIS suite bank; it is a generic PROM that assesses depression, anxiety, physical function, pain interference, fatigue, sleep disturbance, and participation in social roles and activities. |  | Validation is ongoing - some item banks (e.g., fatigue, physical function, pain, emotional distress) have undergone validation - construct validity, reliability according to item response theory. | | | | - | | | | - | | | | Suite of computerized adaptive tests (CATs). Can be administered electronically. Also administered via paper-and-pencil format. |
| **Shortness of Breath** |  |  |  | | | |  | | | |  | | | |  |
| Dyspnoea – 12 (D-12) | D-12 measures the current level of a patient's breathlessness severity, incorporating both physical and affective aspects, and does not depend on activity limitation. The D12 comprises 12 descriptors of breathlessness which are scored by the participant as “None”, “Mild”, “Moderate”, or “Severe”. | 12 | Content validity, construct validity, concurrent validity, factor analysis, test-retest reliability, cross-cultural validation. | | | | Evaluative | | | | 96 | | | | Administered in traditional paper-based format or face-to-face interviews by healthcare professionals or researchers. |
| Modified Medical Research Council (mMRC) Dyspnoea Scale | The mMRC Dyspnoea Scale is used to assess the degree of baseline functional disability due to dyspnoea. It is useful in characterising baseline dyspnoea in patients with respiratory disease. | 5 | Content validity, construct validity, criterion validity, internal consistency, test-retest reliability, large-scale validation. | Descriptive | | | | | | | | | - | | Can be delivered electronically. |
| **Difficulty Sleeping** |  |  |  |  | | | | | | | | |  | |  |
| Pittsburgh Sleep Quality Index (PSQI) | A 19-item questionnaire assessing sleep quality over a month time interval. It consists of several domains, including sleep latency, sleep duration, sleep disturbance, use of sleeping medications, daytime dysfunction, and overall sleep quality | 19 | Internal consistency, test-retest reliability | Evaluative | | | | | | | | | 331 | | Can be delivered electronically. |
| **Headache** |  |  |  |  | | | | | | | | | |  |  |
| Headache Impact Test (HIT) | A 6-item questionnaire that assesses the impact of headaches on daily life activities and functioning. The Headache Impact Test (HIT) is a questionnaire designed to assess the impact of headaches on a person's life. It is used to evaluate the severity of headaches and how they affect a person's ability to carry out their daily activities | 6 | Internal consistency, test-retest reliability | Evaluative | | | | | | | | | | 1662 | Can be administered electronically. |
| **General Pain** |  |  |  |  | | | | | | | | | |  |  |
| Brief Pain Inventory (BPI-SF) | The Brief Pain Inventory - Short Form (BPI-sf) is a 9-item self-administered questionnaire used to evaluate the severity of a patient's pain and the impact of this pain on the patient's daily functioning. | 9 | Content validity, construct validity, concurrent validity, criterion validity, test-retest reliability, internal consistency. | Descriptive/Evaluative | | | | | | | | | | 272 | Can be administered electronically. |
| **Post Traumatic Stress Disorder (PTSD)** |  |  |  |  | | | | | | | |  | | |  |
| Harvard Trauma Questionnaire (HTQ) | The HTQ inquiries about a variety of trauma events, as well as the emotional symptoms considered to be uniquely associated with trauma. | 40 | Content validity, construct validity, concurrent validity, criterion validity, test-retest reliability, internal consistency | Descriptive | | | | | | | | 400 | | | Can be administered electronically. |
| Posttraumatic Stress Disorder Checklist (PCL-5) | The PCL-5 is a 20-item self-report measure that assesses the diagnostic and statistical manual of mental disorders symptoms of PTSD. The PCL-5 has a variety of purposes, including Monitoring symptom change during and after treatment, screening individuals for PTSD, and making a provisional PTSD diagnosis. | 20 | Content validity, construct validity, concurrent validity, criterion validity, test-retest reliability, internal consistency. | | | | | | Evaluative | | | | | 2419 | Can be administered electronically. |
| **Response to Grief** |  |  |  | | | | | |  | | | | |  |  |
| Adult Attitude to Grief Scale (AAGS) | The AAG scale was devised as a tool to test both the validity of the grief reactions – overwhelmed feelings and controlled functioning – and the resilient capacity to balance these elements. The AAG consists of nine items on a five-point Likert scale, from strongly agree (score 4), to strongly disagree (score 0). | 9 | Reliability analysis, factor analysis (content validity, construct validity, concurrent validity), and test-retest reliability. | | | | | | Descriptive | | | | | - | Can be administered electronically. |
| **Inventory of Complicated Grief (ICR)** | The ICG is a widely recognized PROM used to assess complicated or prolonged grief reactions. It consists of 19 items that ask respondents to rate their experiences and feelings related to the loss of a loved one. This tool helps identify individuals who may be at risk for complicated grief and may benefit from clinical intervention. | 19 | Reliability analysis, factor analysis (content validity, construct validity, concurrent validity), and test-retest reliability. | | | | | Evaluative | | | | | | - | Can be administered electronically. |

**Search Sources**

- PubMed/MEDLINE
- Web of Science
- Scopus
- APA PsycINFO
- Google Scholar
- Australian Commission on Safety and Quality in Healthcare databases.

**Criteria for Data Extraction**

These are a list of specific criteria used to extract relevant information from each included study or document. These criteria ensure consistency and objectivity in the extraction process.

• Mode **of Administration**: How the PROM instrument is administered (e.g., paper, electronic).

• Purpose: The intended goal or focus of the PROM instrument (e.g., assessing symptoms, functional status).

• Participants: Number of participants involved in initial studies or validation.

• **Average Administration Time**: Typical duration required for a participant to complete the instrument.

• **Original Language of Validation**: Language in which the PROM instrument was initially validated.

• **Domains**: List of domains or areas of measurement covered by the PROM instrument (e.g., physical functioning, emotional well-being).

• **Reliability:** Measures of internal consistency (e.g., Cronbach’s alpha), and test-retest reliability (e.g., ICC).

• **Validity:** Evidence supporting validity, including face validity, content validity, criterion validity, and construct validity.

• **Cost:** Any information regarding the cost implications of using the instrument.

• **Resources Needed**: Resources required to administer and manage the tool.Bottom of Form
